# 

**Published:** 1854-04

**Authors:** 


Our next number will contain articles from Prof. Lee, Dr. W. C. Butler, and "Rusticus," together with Mr. Germain's speech on the Dissection Bill. We hope that the favors extended to us by able correspondents will be continued. We should like to make the Journal, as far as possible, the expression of medical opinion and sentiment, not of Western New York only, but of the entire range of country included in our mail-list.

---

The Summer Course of Lectures and Recitations at the Buffalo Medical College commences on the first Monday in April.

